# Origin of unusual spinel-to-layered phase transformation by crystal water†
†Electronic supplementary information (ESI) available: Calculation details, intercalation geometries, interlayer distance. See DOI: 10.1039/c7sc04114d


**DOI:** 10.1039/c7sc04114d

**Published:** 2017-10-24

**Authors:** Eunjeong Yang, Heejin Kim, Sangryun Kim, In Kim, Jaehoon Kim, Hyunjun Ji, Jang Wook Choi, Yousung Jung

**Affiliations:** a Graduate School of EEWS , Korea Advanced Institute of Science and Technology , 291 Daehak-ro , Daejeon , 34141 , Republic of Korea . Email: ysjn@kaist.ac.kr; b Electron Microscopy Research Center , Korea Basic Science Institute , 169-148 Gwahak-ro , Daejeon 34133 , Republic of Korea; c Institute for Materials Research , Tohoku University , Sendai 980-8577 , Japan; d School of Chemical and Biological Engineering , Institute of Chemical Processes , Seoul National University , 1 Gwanak-ro , Seoul 08826 , Republic of Korea . Email: jangwookchoi@snu.ac.kr

## Abstract

It is well known that many layered transition metal oxides can transform into a spinel structure upon repeated battery cycling, but a phase transition in the opposite direction is rare. Recently, the transformation from spinel Mn_3_O_4_ to layered MnO_2_ was observed during the operation of a Mg battery in aqueous conditions, resulting in high performance Mg batteries. We hereby use *ab initio* calculations to unveil the mechanism by which crystal water plays a critical role in this unique transformation. Once inserted into the spinel form, a water molecule donates an electron, offering a key structural and thermodynamic driving force to initiate the transformation process. These crystal water molecules then get favorably clustered into a planar form in the layered structure and act as a stabilizing agent for birnessite. Kinetically, the inserted crystal water dramatically promotes the necessary rearrangement of Mn during the transition by lowering the activation barrier by >2 eV. The present structural, thermodynamic and kinetic understanding of the crystal water-driven phase transition provides novel insights to further the design of related low dimensional hydrated materials for multi-valent cathodes.

## Abstract 2

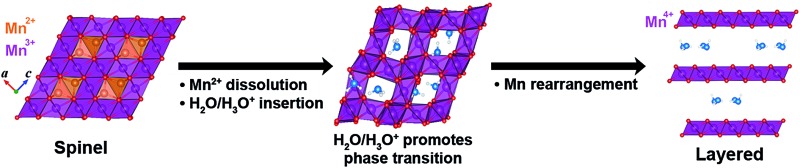
Crystal water mediated phase transition: the underlying thermodynamic and kinetic role of crystal water is investigated using *ab initio* calculations.

## Introduction

Layered transition metal (TM) oxides have been widely investigated as electrode materials for both lithium and post-lithium ion batteries, since they potentially have a higher energy density than other materials including sulfides, fluorides and polyanionic compounds.^1^–^3^ Birnessite-type manganese oxides, or birnessites, are naturally abundant compounds with a layered structure and the general chemical formula AMn_2_O_4_·(H_2_O)_*m*_, where A can be various cations such as Na^+^, K^+^, Mg^2+^ or Ca^2+^.^4^ Each layer of the birnessite is composed of edge shared MnO_6_ octahedra and the interlayer space is filled with pillaring cations and/or water molecules. This Mn-based layered material is appealing in terms of its relatively simple synthesis procedure, cost-efficiency and non-toxic nature, along with its capability of incorporating alkali ions *via* two-dimensional diffusion. Therefore, both the electrochemical insertion of Li, Na or Mg into birnessites and the pseudocapacitive charge storage of birnessites have been investigated.^5^–^10^

Recently, a birnessite cathode material was successfully synthesized from spinel Mn_3_O_4_ precursors using an electrochemical method.^11^,^12^ When typical electrochemical charge and discharge cycles were performed in aqueous electrolytes, the structural transformation of a 3-dimensional spinel to a 2-dimensional birnessite, *i.e.* a spinel-to-layered transformation, was observed. Even though it has long been known that many layered TM oxides transform into a spinel structure, *i.e.* a layered-to-spinel transformation, upon repeated battery cycling,^13^–^18^ the phase transition in the opposite direction that takes place during the formation of birnessite is unusual. The main driving force for this spinel-to-layered structure conversion is suggested to be the insertion of crystal water on the basis of experimental data which showed that the phase transition occurs only when spinel Mn_3_O_4_ is cycled in aqueous electrolytes^11^,^12^,^19^ and that a higher water content in the compound resulted in a more efficient transformation into birnessite,^20^ which makes this spinel-to-layered transformation even more peculiar. Additionally, Na- or Mg-containing birnessite shows improved capacity retention and rate capability over repeated battery cycling when compared to the anhydrous sample.^10^,^21^ Such enhancements in the energy and power density due to structural water molecules have also been reported in tungsten oxides^22^ and vanadium oxides.^23^ Even so, the beneficial effects of crystal water molecules in both enhancing the electrochemical performance and mediating the spinel-to-layered phase transition have not yet been fully understood.

Herein, in an effort to broaden our understanding of birnessites and the possibilities of similar hydrated materials as electrode materials, we investigate the spinel-to-birnessite transformation on the basis of first-principles calculations, focusing on the effect of crystal water on this structural transition.

## Results and discussion

### Intercalation of crystal water and alkali ions

**Fig. 1:**
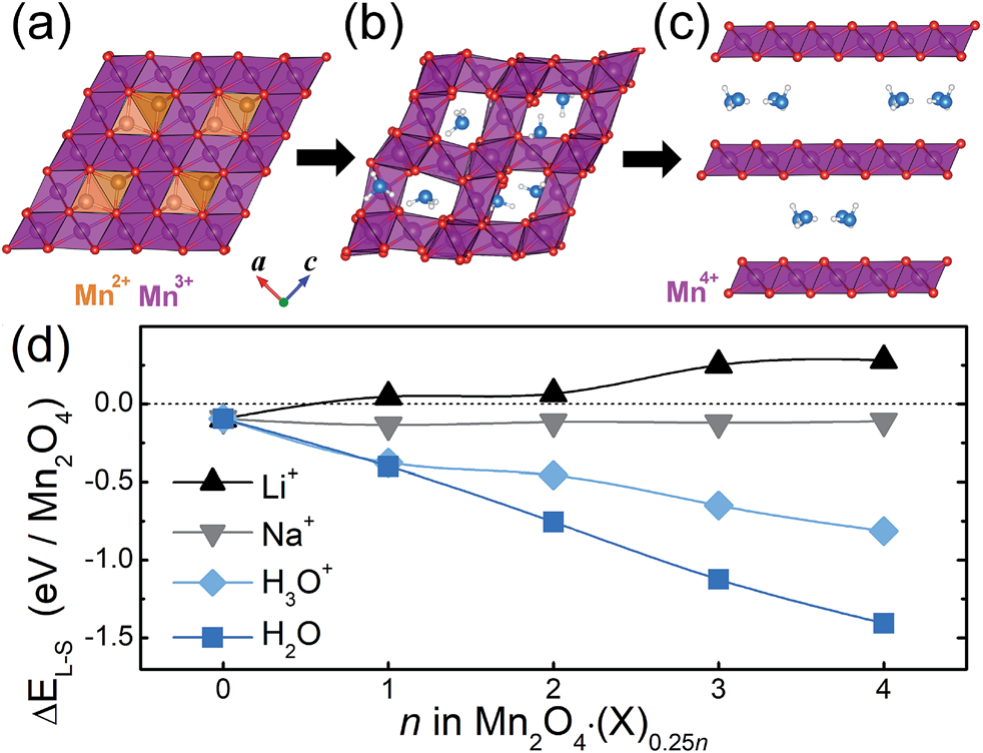
(a–c) A scheme of the spinel-to-layered phase transition: (a) spinel Mn_3_O_4_, (b) water intercalated Mn_2_O_4_ after the Mn^2+^ dissolution, (c) transformed layered Mn_2_O_4_ with interlayer H_2_O molecules. The blue and white spheres are the O and H of the crystal water, respectively. Mn^2+^ cations are orange and the purple octahedrons are the Mn_2_O_4_ framework. (d) The stability of the layered structure relative to that of the spinel structure, defined as Δ*E*_L–S_ = *E*_Layered_ – *E*_Spinel_ for Mn_2_O_4_·(X)_0.25*n*_, where X = H_2_O, H_3_O^+^, Li^+^ or Na^+^.

The chemical formula of spinel Mn_3_O_4_, which belongs to the space group *I*4_1_/*amd*, can be rewritten as Mn^2+^Mn_2_^3+^O_4_, where the Mn^2+^ and Mn^3+^ cations occupy the tetrahedral and octahedral sites respectively (Fig. 1a). When we compared the stability of the octahedral and tetrahedral sites by removing one Mn atom from each site, tetrahedral Mn was less stable than octahedral Mn by 60 meV per formula unit, this is consistent with experimental measurements in which Mn in the tetrahedral site was preferentially dissolved over the octahedral Mn.^19^ Crystallographically speaking, since the spinel structure shares the same close-packed oxygen framework as the layered structure,^24^ the phase transition between the two structures is fairly feasible without changing the oxygen stacking sequence. For this reason, it is well known that layered lithium manganese oxide (Li_1–*x*_MnO_2_) spontaneously transforms to the spinel structure (LiMn_2_O_4_) upon battery cycling, just by migrating the transition metal into the Li layer;^13^,^25^,^26^ the phase transition that occurs when preparing birnessite is exactly the opposite process.

A scheme of the spinel-to-layered phase transition, with the help of crystal water molecules, is summarized in Fig. 1. According to recent studies on the electrochemical generation of Mg birnessite, the spinel-to-layered transition process is initiated by the dissolution of Mn^2+^ from the spinel Mn_3_O_4_ (Fig. 1a),^20^ which leaves the chemical composition of the spinel compound as Mn_2_O_4_. The water molecules then intercalate into the vacant space in the remaining Mn_2_O_4_ framework (Fig. 1b). During this intercalation of water molecules and subsequent phase transition, the tetrahedral sites and the octahedral sites between them play an important role, since they can potentially accommodate diverse guest species and they also sit in the ionic migration channel required for the phase transition. Here, we considered H_2_O and H_3_O^+^ as the intercalation form of crystal water, since it is suggested they both participate in the overall phase transition process on the basis of thermal and electron microscopy analyses. That is, albeit there is a low concentration of H_3_O^+^ under neutral pH conditions, its insertion into the spinel structure is detected, while the final form of crystal water in the layered birnessite is H_2_O.^19^

From extensive calculations, we found that at most one water molecule can be intercalated into the Mn_2_O_4_ spinel framework without disrupting the structure, which corresponds to the chemical composition MnO_2_·(X)_0.5_, where X = H_2_O or H_3_O^+^. Detailed intercalation geometries are presented in Fig. S1 (ESI).† Once inserted, the H atom of the water molecule (H^w^) forms O–H^w^ bonds with the O atoms of the spinel framework, where the O–H^w^ distance is 1.30–1.57 Å. Thus the O atom of the water molecule (O^w^) is placed off-center in the tetrahedron or octahedron as shown in Fig. 1b and S1.† In this stage, the framework is still securely sustained by the octahedral Mn pillars, leaving the interplanar distance of the (101) plane to be 4.8–5.2 Å regardless of the water content (Fig. S2†). This interplanar distance is consistent with the experimental value.^19^ Lastly, the inserted crystal water promotes the phase transformation from the spinel to the layered structure with an increased (101) interplanar distance of 7.27 Å for X = H_2_O (Fig. 1c), the crystal water molecules are located in the center of the interlayer space as experimentally observed.^12^,^20^ In the birnessite, the interlayer distance for X = H_3_O^+^ (6.77 Å) is quite a bit smaller than the observed value (7.2 Å),^20^ confirming that the final form of crystal water in the birnessite is H_2_O.

In order to investigate whether this water-inserted spinel phase thermodynamically favors the formation of the birnessite structure, we evaluated the energy difference between the spinel (Fig. 1b) and layered (Fig. 1c) Mn_2_O_4_·(X)_0.25*n*_ structures, defined as Δ*E*_L–S_ = *E*_Layered_ – *E*_Spinel_, where *E*_Layered_ and *E*_Spinel_ correspond to the calculated total energy of each structure. The calculated Δ*E*_L–S_ values, when varying *n* from 0 to 4 in Mn_2_O_4_·(X)_0.25*n*_, are compared in Fig. 1d for X = H_2_O, H_3_O^+^, Li^+^ and Na^+^. Here, we assumed that all systems are neutral since the reaction is electrochemical and the electrons are supplied externally for charge neutrality. It is noted that the systems containing crystal water molecules, *i.e.* X = H_2_O or H_3_O^+^, have a thermodynamic driving force (negative Δ*E*_L–S_ values) for the transformation into the layered or birnessite structure. Moreover, the degree of preference for the layered form increases as the amount of water molecules (*n*) in the compound increases. This is in good agreement with a previous experimental report suggesting that a higher crystal water content promotes an efficient phase transition into birnessite.^20^ In the same literature, it was also pointed out that preventing the insertion of alkali ions facilitates the spinel-to-layered transformation and this is consistent with the present result, which indicates that the Δ*E*_L–S_ values for X = Li and Na are close to zero or even positive (no thermodynamic driving force for such a phase transition). In addition, the calculated energetic preference for a layered structure increases in the order of Li < Na < H_3_O^+^ < H_2_O insertion, which is identical to the order of increasing degree of the spinel-to-birnessite phase transformation observed in experiments.^20^

### Interaction between the crystal water and Mn framework

**Fig. 2:**
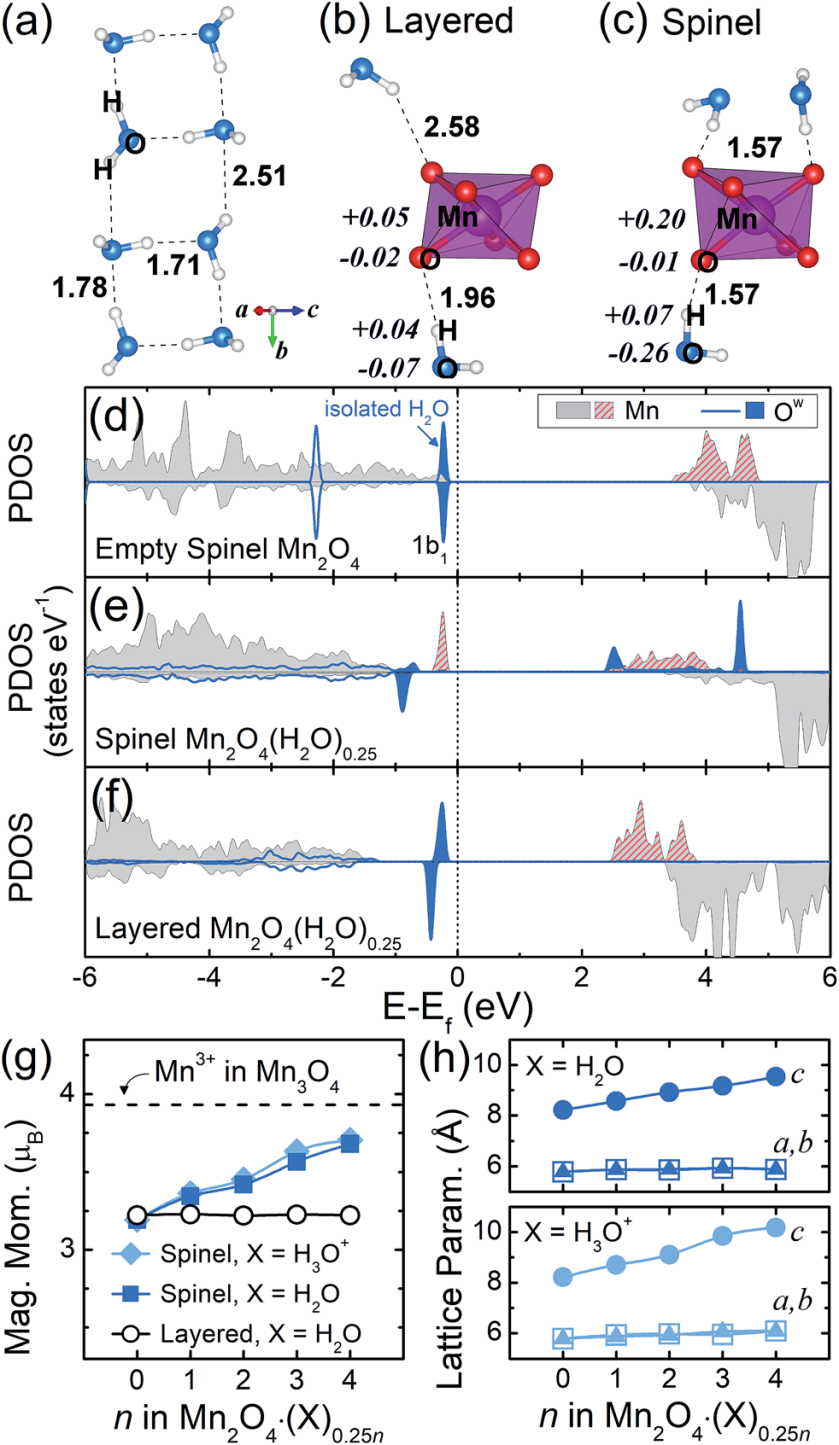
(a) A water cluster formed in the layered Mn_2_O_4_·(H_2_O). The numbers next to the dotted lines in bold denote the distance. (b and c) MnO_6_ octahedra and nearby crystal water molecules for the (b) layered and (c) spinel Mn_2_O_4_·(H_2_O). The italicized numbers are the variation in charge density and the unitalicized numbers are bond distances. The charge densities are integrated by the Bader method. (d–f) The PDOS of (d) empty spinel Mn_2_O_4_, (e) spinel Mn_2_O_4_·(H_2_O)_0.25_ and (f) layered Mn_2_O_4_·(H_2_O)_0.25_. The upper and lower halves of each panel are spin up (majority) and down, respectively. The isolated H_2_O is presented together in (d). (g) The magnetic moments of Mn in the spinel and layered Mn_2_O_4_·(X)_0.25*n*_. The value for Mn^3+^ in Mn_3_O_4_ (3.93) is presented with a dashed line for comparison. (h) The change in lattice parameters in spinel Mn_2_O_4_·(X)_0.25*n*_.

First of all, as presented in Fig. 1c and 2a, we found that H_2_O molecules in the layered structure form a planar water cluster. According to a previous study, the growth of a small water cluster is favored by enthalpy, while it is unfavored by entropy; thus, at an ambient temperature where the entropic term is significant, the development of water clusters was exothermic.^27^ This result suggests that a 2D confined space, like our interlayer space, can constrain the motion of water and thus can promote the formation of water clusters. Recently, square-like ice was observed in graphene nanocapillaries, in which the authors propose that such clusters could develop in general when a 2D confined space is hydrophobic,^28^,^29^ since too strong an interaction between the wall and water can hinder the clustering of water. The latter point suggests that, in our layered system, the interaction between H_2_O and the layered Mn_2_O_4_ framework is as small as the one in a hydrophobic channel. Indeed, in the layered Mn_2_O_4_·(H_2_O), the interaction energy between H_2_O and the framework is only 0.015 eV per H_2_O, whereas the interaction among H_2_O molecules is 0.41 eV per H_2_O. This weak interaction between H_2_O and the framework is also reflected in the long distance and marginal charge transfer between them, as shown in Fig. 2b.

On the other hand, crystal water molecules in the spinel Mn_2_O_4_·(X)_0.25*n*_ interact strongly with the spinel framework and reduce the Mn. In the case of X = H_3_O^+^, intercalated hydronium molecules form a O–H^w^ bond (distance of 1.3 Å) with a nearby MnO_6_ polyhedron, in which the corresponding electron for charge neutrality reduces the Mn from 4+ to 3+ in the Mn_2_O_4_ framework. Surprisingly, we found that the inserted neutral H_2_O molecule also reduces the Mn_2_O_4_ framework by donating electrons. In the latter case, each of the MnO_6_ polyhedra is surrounded by H_2_O molecules with an O–H^w^ bond length of 1.57 Å (Fig. 2c); this value is in between those of typical covalent (∼1 Å) and hydrogen (∼2 Å) bonds, which reflects that the charge transfer between the framework and H_2_O is substantial. When compared to isolated H_2_O molecules, each H_2_O in the spinel structure donates approximately 0.2 electrons to the interacting MnO_6_ units and as a result, Mn atoms in the framework accept electrons from the water molecules and thus are reduced.

This charge transfer between H_2_O and the framework can be seen clearly from the projected density of states (PDOS) of the O^w^-2p and Mn-3d states of the spinel and layered compounds (Fig. 2d–f). The single point calculation with a PBE0 functional^30^,^31^ is employed for drawing the PDOS using GGA + U geometries. The highest occupied molecular orbital (HOMO) of an isolated water molecule is 1b_1_ as denoted by the blue area in Fig. 2d. When water is inserted into the spinel Mn_2_O_4_ (Fig. 2e), the 1b_1_ orbital loses most of its spin up electrons, resulting in a significant number of unoccupied states above the Fermi level. The electron density of the isosurfaces (Fig. S3†) confirm that these unoccupied O^w^ states are originally 1b_1_ orbitals. In contrast, the layered Mn_2_O_4_·(H_2_O)_0.25_ does not show such a drastic oxidation of water molecules (Fig. 2f). Furthermore, the empty spinel Mn_2_O_4_ has an antibonding orbital at the lowest unoccupied state which is composed of Mn spin up (see the crystal orbital Hamilton population^32^ in Fig. S4†). This antibonding orbital shifts below the Fermi level when crystal water is inserted (red hatching in Fig. 2e), indicating that the Mn is reduced by the crystal water insertion. Therefore, the evidence from the PDOS clearly points to a conclusion that a charge transfer, from the crystal water to Mn in the spinel structures, causes the reduction of Mn. We note, from the methodological point of view, that the GGA + U functional gives essentially the same charge transfer results as PBE0, as shown in Fig. S5.†

The change in oxidation state of Mn caused by the insertion of crystal water can be more evidently seen from the magnetic moment that is proportional to the number of unpaired electrons. Fig. 2g displays the net magnetic moments of Mn_2_O_4_·(X)_0.25*n*_ compounds with the spinel and layered structures. We note that the net magnetic moments are generally underestimated in oxide materials since electrons are polarized/delocalized from the metal center to neighboring oxygen.^33^,^34^ When there is no crystal water (*n* = 0), the magnetic moments are around 3.2 for all the Mn structures regardless of the crystal structure, and these values are close to the number of unpaired electrons for Mn^4+^, which is 3. However, when *n* > 0 (*i.e.* with crystal water molecules), the magnetic moments have different values depending on which structure the Mn_2_O_4_·(X)_0.25*n*_ takes. For the spinel structure, when *n* = 4 for example, the average magnetic moment of Mn increases to 3.68 and 3.70 for X = H_2_O and H_3_O^+^, respectively; this implies that Mn atoms in these compounds were reduced toward the 3+ state due to the crystal water molecules. For the layered Mn_2_O_4_·(H_2_O)_0.25*n*_, on the other hand, the magnetic moments of all the Mn species have the same value (3.2) for all *n*, indicating that the oxidation state of Mn^4+^ in the layered compound remains unchanged with varying crystal water content; this result is attributed to the negligible interaction between the framework and crystal water in the layered structure as discussed above. As a result of the reduction of Mn from 4+ to ∼3+ in the presence of water molecules, the cell parameters of the spinel Mn_2_O_4_·(X)_0.25*n*_ compounds increase anisotropically (Fig. 2h) since the Mn^3+^ state is susceptible to a Jahn–Teller distortion. We note that the change in the distance between the (101) planes is marginal (less than 0.2 Å), as observed experimentally,^10^ since the Jahn–Teller distortion arises along the *c*-direction and this leads to a sliding of the Mn layers. The distortion index (*D*), defined by Baur,^35^ also supports the reduction of the Mn center: the *D* value of the spinel Mn_2_O_4_·(X)_1.0_ is 0.035 and 0.067 for X = H_2_O and H_3_O^+^, respectively, which are between that of the Mn^3+^ case (0.074 in Mn_3_O_4_) and Mn^4+^ case (0.002 in layered Mn_2_O_4_·(H_2_O)_1.0_).

### Thermodynamic contributions to the phase transition

**Fig. 3:**
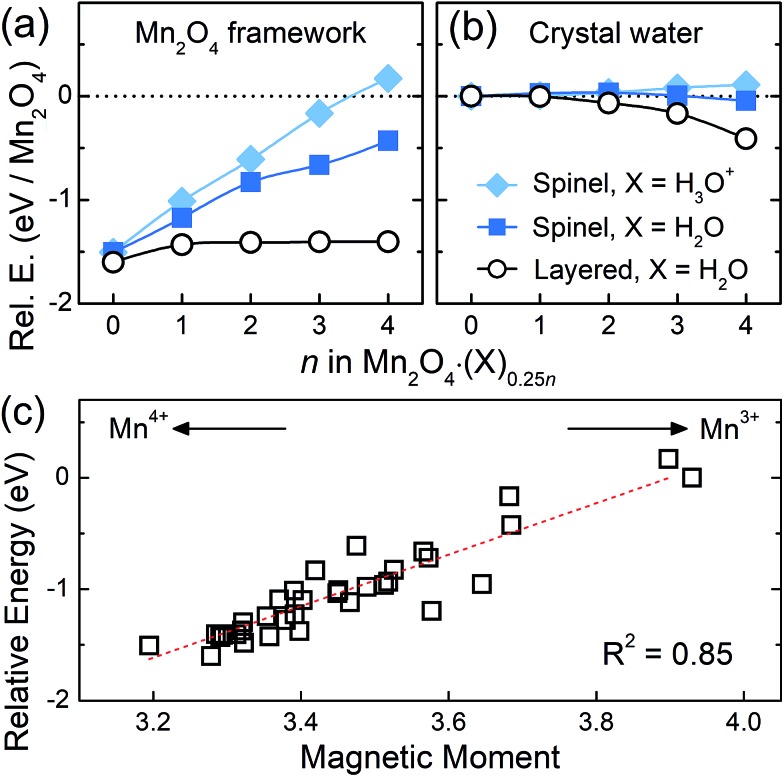
The relative energies of the (a) Mn_2_O_4_ frameworks and (b) crystal water molecules for each composition of spinel and layered Mn_2_O_4_·(X)_0.25*n*_. (c) The correlation between the magnetic moment of the Mn center that indicates its oxidation state and the relative energy of the Mn_2_O_4_ framework. A more negative *y* value indicates a more stable structure.

On the basis of these analyses of the interaction between crystal water and the Mn framework, the increasing stability of the layered structure upon water intercalation can be further understood by breaking down the energetics into the contributions from the framework and crystal water. For that, we evaluated the single point energy of the Mn_2_O_4_ framework and intercalated X molecules, whose geometries were taken from Mn_2_O_4_·(X)_0.25*n*_. Then we compared their energies to those of the initial Mn_2_O_4_ framework (single point energy of the Mn^2+^ dissolved structure taken from Mn_3_O_4_) and geometry-optimized isolated X molecules, which are referenced as zero in Fig. 3a and b, respectively. While the crystal water is H_2_O in the transformed birnessite, its initial form can be both H_2_O and H_3_O^+^ in the spinel as discussed above; thus we compared the layered Mn_2_O_4_·(H_2_O)_0.25*n*_, spinel Mn_2_O_4_·(H_2_O)_0.25*n*_ and spinel Mn_2_O_4_·(H_3_O^+^)_0.25*n*_ structures. Overall, it is clear that the spinel and layered phases exhibit entirely opposite behaviors. The spinel structure is destabilized predominantly due to the framework distortion, while the stability of the water molecules in it remains the same regardless of water content. In contrast, the framework stability of the layered structure is almost unaffected by the insertion of crystal water, but the stability of the intercalated water molecules substantially increases with increasing water content. These phenomena observed in Fig. 3a and b are essentially driven by the crystal water, which will be discussed below.

In Fig. 3a, the Mn_2_O_4_ frameworks of the structures that contain water molecules are more stable (negative *y* values) when compared to the initial Mn_3_O_4_ compound (dotted zero line). The empty Mn_2_O_4_ framework (*n* = 0) is the most stable since it is the relaxed geometry at a given Mn_2_O_4_ composition. With an increasing water content in the spinel crystal (*n*), more Mn reduces from 4+ to 3+, where the former and latter are Jahn–Teller inactive and active species, respectively; this enlarged distortion during the redox reaction imposes a substantial energy penalty and thus destabilizes the spinel system. For the same reason, the Mn_2_O_4_ framework of the initial Mn_3_O_4_ is the most unstable, since all the framework Mn atoms are Mn^3+^ in Mn_3_O_4_. When we draw the relative stability of the Mn_2_O_4_ framework *versus* the oxidation state of Mn for all the structures considered in this work (a total of 35 frameworks as described in the ESI†), a linear correlation is reasonably clear (*R*^2^ = 0.85), supporting the idea that this destabilization is caused by Mn^3+^. In the Mn_3_O_4_, spinel Mn_2_O_4_·(X)_0.25*n*_ and layered Mn_2_O_4_·(H_2_O)_0.25*n*_ structures, the Mn atoms that compose the Mn_2_O_4_ framework have the oxidation state of 3+, 3–4+ (between 3+ and 4+) and 4+, respectively, as identified in oxidation state analyses. Therefore, the overall stability of the framework, which is inversely proportional to the number of Mn^3+^, is in the increasing order of Mn_3_O_4_ < spinel Mn_2_O_4_·(X)_0.25*n*_ < layered Mn_2_O_4_·(H_2_O)_0.25*n*_. This stabilization of the layered framework results in the primary driving force for the spinel-to-layered phase transition. We note that Li and Na inside the spinel can also reduce the Mn center. However, unlike H_2_O, the Li or Na reduces the Mn to 3+ in the layered counterpart as well, due to its high ionicity. Therefore, the stability of the layered form with Li or Na is lower than the stability of the same structure with H_2_O, leading to a smaller net thermodynamic driving force for the transition to a layered phase as shown in Fig. 1d.

In Fig. 3b, strong water–water interactions are observed in the layered structure with increasing water content, due to the aforementioned clustering of water molecules. This leads to charge reallocation within crystal water clusters, while the charge transfer between the layered Mn_2_O_4_ framework and H_2_O molecules is marginal (0.03 electrons as shown in Fig. 2b) and thus, once transformed to the 2D birnessite, the layered framework is almost unaffected by the amount of H_2_O insertion, as shown in Fig. 3a. To summarize, the crystal water itself contributes to the stability of the layered structure by clustering; but, in addition, it destabilizes the spinel framework by chemically reducing the Mn of the spinel structure, providing a thermodynamic driving force for the transition to layered birnessite.

### Kinetic role of crystal water in the phase transition

**Fig. 4:**
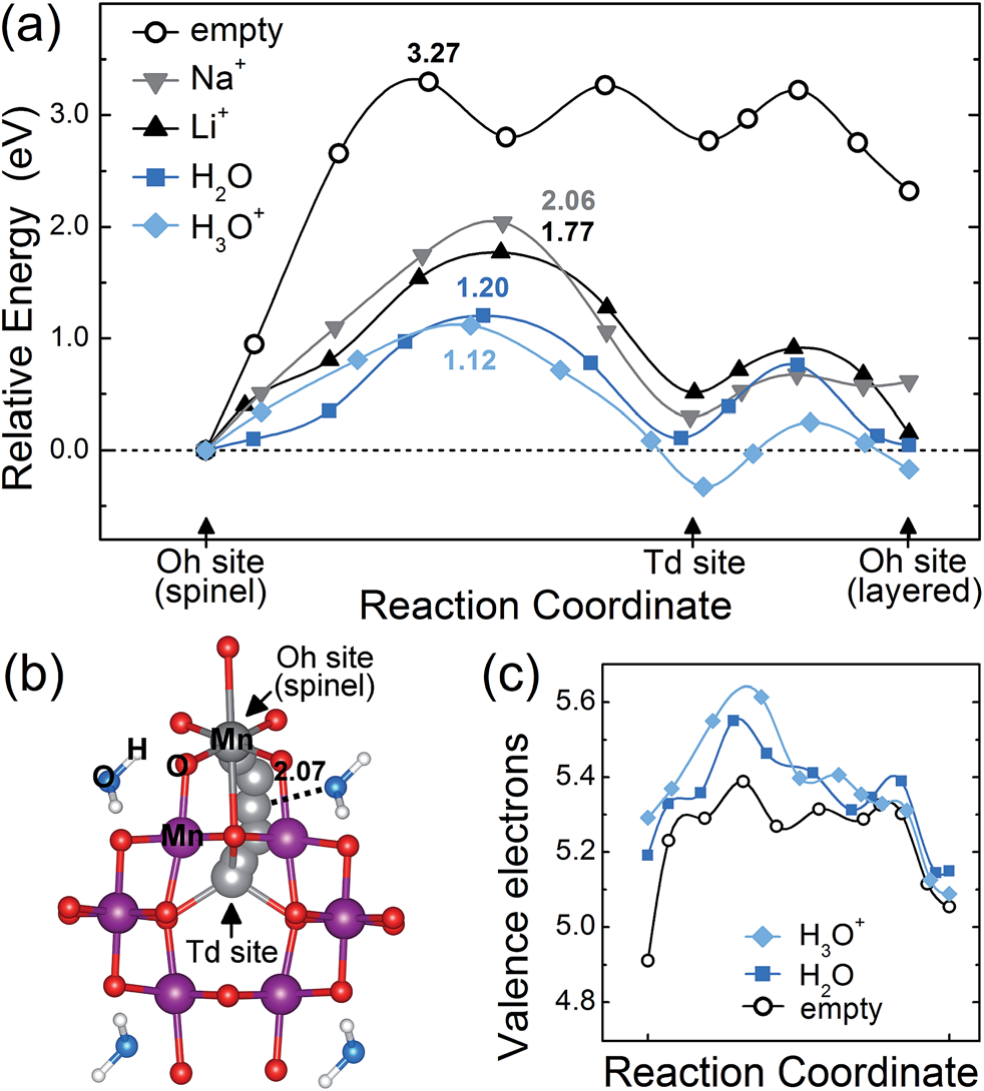
(a) The calculated energy barriers for Mn migration in spinel Mn_2_O_4_·(X)_0.25*n*_ for *n* = 0 (empty) and *n* = 2 (X = Na^+^, Li^+^, H_2_O and H_3_O^+^). The numbers are migration barriers. (b) The traveling pathway of target Mn (gray sphere) from an Oh site to a Td site in the presence of crystal water. The number denotes a distance. (c) The charge density of the traveling Mn at each intermediate migration position.

Lastly, in order to investigate the effect of crystal water on the spinel-to-layered transition from a kinetic perspective, we evaluated the energy barriers for Mn migration in spinel Mn_2_O_4_·(X)_0.25*n*_ with and without crystal water molecules. The results are depicted in Fig. 4a and b. Since the phase transition requires part of the Mn in the Mn_2_O_4_ framework to be rearranged, a single Mn atom in the spinel framework was migrated from one octahedral site to another through the nearest tetrahedral site, so that a reverse procedure of the known layered-to-spinel transformation can be modelled. When there is no crystal water in the system (*n* = 0; empty), the calculated activation barrier for Mn migration is 3.27 eV. This energy barrier is reduced significantly to 1.20 eV (1.12 eV) in the presence of H_2_O (H_3_O^+^) molecules, indicating that the crystal water greatly assists the diffusion of Mn for structural transformations.

The above results can be explained by understanding the structural and electronic aspects of the material. First, the lattice parameters and cell volume are overall expanded with the insertion of water as shown in Fig. 2h, which enlarges the diffusion channel for Mn migration. Second, Mn atoms have 4+ oxidation states without crystal water, whereas they are in ∼3+ states with crystal water. The electron density analysis (Fig. 4c) clearly shows that the presence of water molecules leads to a higher electron density (reduced oxidation state) for the traveling Mn atom, which decreases the electrostatic repulsion from neighboring atom centers more effectively. Third, the Mn migration process without the presence of water generates 1–3 broken bonds and the corresponding partially unpaired electrons destabilize the system. When intercalated species such as crystal water and alkali ions are in the framework, however, the water or alkali ions can stabilize the intermediate Mn states with broken bonds by accepting some of their unpaired electrons, and thus reduce the migration barrier. Due to these aspects, the intercalated Na and Li ions, despite their positive charge, also decrease the Mn migration barrier to 2.06 and 1.77 eV, respectively, although they are less effective than crystal water. When examining the Mn migration pathway in Fig. 4b, the traveling Mn inclines toward a water molecule and makes a Mn–O^w^ bond with a distance of 2.07 Å. This partially-bonded intermediate state, which is not observed in the cases with Li or Na inserted (Fig. S6†), additionally lowers the Mn migration barrier.

## Conclusions

We revealed the underlying mechanism of how crystal water plays an essential role in the unusual spinel-to-layered phase transformation that takes place during the electrochemical cycling of Mn_3_O_4_ in aqueous media. We found that spinel Mn_3_O_4_ compounds energetically prefer a layered structure after Mn^2+^ dissolution when crystal water is present. Electronically, the inserted crystal water destabilizes the spinel Mn_2_O_4_ framework by reducing Mn^4+^ to distortive Mn^3+^, whereas only a weak interaction between crystal water and layered Mn_2_O_4_ exists, leaving Mn as stable Mn^4+^ in the layered framework. Instead, water molecules form a planar cluster in the 2D confined space of the layered structure, further stabilizing the layered phase compared to the spinel phase, in which such water clustering is not feasible. This difference in water–framework interactions between the spinel and layered phases provides a key structural and thermodynamic driving force for the experimentally observed spinel-to-layered transition. Kinetically, the crystal water considerably lowers the activation barrier for the Mn migration needed for the phase conversion. The present structural, energetic and kinetic aspects elucidating the role of crystal water undoubtedly provide critical insight that is valuable for understanding and designing robust hydrated 2D materials for various applications.

## Conflicts of interest

There are no conflicts to declare.

## Supplementary Material

Supplementary information
