# Supplementary material for: Origin of unusual spinel-to-layered phase transformation by crystal water
Source: Chem Sci. 2017 Oct 24;9(2):433–8. doi: 10.1039/c7sc04114d (PMC5868318; doi:10.1039/c7sc04114d)
Supplement: Supplementary file 1 [file SC-009-C7SC04114D-s001.pdf]

Electronic Supplementary Material (ESI) for Chemical Science.
This journal is © The Royal Society of Chemistry 2017

Electronic Supporting Information

Origin of Unusual Spinel-to-Layered Phase Transformation by Crystal Water

Eunjeong Yang,[a,†] Heejin Kim,[b,†] Sangryun Kim,[c] In Kim,[a] Jaehoon Kim,[a] Hyunjun Ji,[a] Jang Wook Choi[a,d,*] and Yousung Jung[a,*]

[a] *Graduate School of EEWS, Korea Advanced Institute of Science and Technology, 291 Daehak-ro, Daejeon, 34141, Republic of Korea.*
[b] *Electron Microscopy Research Center, Korea Basic Science Institute, 169-148 Gwahak-ro, Daejeon 34133, Republic of Korea.*
[c] *Institute for Materials Research, Tohoku University, Sendai 980-8577, Japan.*
[d] *School of Chemical and Biological Engineering and Institute of Chemical Processes, Seoul National University, 1 Gwanak-ro, Seoul 08826, Republic of Korea*
[e] *† These authors contributed equally to this work.*

Experimental Section.

Calculation details. We performed spin polarized calculations using the Perdew-Burke-Ernzerhof (PBE) functional and projector-augmented wave (PAW) method as implemented in the VASP software.[1-3] An energy cutoff of 520 eV was used as a basis set, and the Monkhorst-Pack k-point mesh was sampled with a spacing of <0.03 A^{-1}.[4] We applied the U correction in a rotationally invariant form to address the self-interaction of d-electrons in Mn (U_{eff} = 4.0).[5-7] To include a dispersion interaction in considered system, the Grimme's D3 method with zero damping was employed.[8] While we used the D3 parameters optimized for PBE functional (S_6 = 1.0, S_R = 1.217, and S_8 = 0.722), the geometries and energetics obtained with PBE+U+D3 were comparable to the D3 corrected PBE0 method as shown in Table S1. For the PBE0 calculations, the D3 parameters of S_6 = 1.0, S_R = 1.287, and S_8 = 0.928 were used as tested by Grimme and co-workers (reference 31 in the text). Since the energy differences between ferromagnetic (FM) and antiferromagnetic (AFM) orderings are less than 0.1 eV (which is much smaller than the energy difference between spinel vs. layered phases by an order of magnitude), and the lowest energy magnetic structure is altered depending on the number of intercalated ions (Fig. S7), we assumed FM ordering for all calculations to minimize the unnecessary complications and potential artifacts arising from magnetic ordering. To obtain the ground structure, all geometries were fully relaxed until the remaining forces were less than 0.01 eV A^{-1}. The Bader analysis was used to obtain charge density and magnetic moment values; for these calculations, denser grid was used.[9] To estimate the energy barriers associated with Mn migration, we used the climbing-image nudged elastic band (CI-NEB) method in the 2×2×1 supercells.[10] To calculate the positively charged systems containing H_3O^+ ion (Fig. S1), we changed the total number of electrons in the system.

Li and Na intercalation geometry. To obtain the insertion structure of Li and Na in the spinel Mn_2O_4, we used the Mn^{2+} dissolved Mn_3O_4 as a starting geometry, which contains 8 Mn and 16 O atoms. This

empty spinel structure has 4 tetrahedral sites (4*a*) and 8 octahedral sites (8*c*) per unit cell, which are able to accommodate the Li or Na. We prepared all possible Li or Na intercalation orderings at these empty sites with symmetric considerations; and then we performed full geometry relaxation. As a result, both Na and Li atoms prefer the tetrahedral site by -0.22 and -0.42 eV per formular unit, respectively, compared with each octahedral site.

Water intercalation geometry in the spinel structure. For the H_2O and H_3O^+ intercalation in the spinel structure, similar approach is adopted; that is, first we placed the O^w atom at the center of octahedral or tetrahedral site as the same way as Li or Na. Next, to determine the initial position of H^w atoms, we assumed that H^w atoms are located in the vertex direction of octahedron or tetrahedron to form hydrogen bonds with the O of spinel framework. Then, there are 6 and 4 possible H^w positions for each octahedral and tetrahedral sites, respectively. If the H^w occupies 2 (3) positions among total 6 or 4 positions, the inserted molecule will be the H_2O (H_3O^+). After setting the initial position of H_2O or H_3O^+ in above manner, we fully relaxed the atomic position and lattice parameters.

Water intercalation geometry in the layered structure. The initial position of crystal water in layered structures was determined by placing O^w in octahedral sites between Mn_2O_4 layers. For that, we considered all possible configurations for a given composition (e.g. 70 configurations for n = 4 in $Mn_2O_4{\cdot}(X)_{0.25n}$). Subsequently, we performed geometry optimization and the energetically most stable configuration was chosen for further analyses.

Details on the Fig.S1. First of all, we considered both H_3O^+ and H_2O as the intercalation form of water molecules since both were observed in experiments, albeit a low concentration of H_3O^+ in neutral pH condition. We suspect that the generation of proton on the surface of manganese oxide

could be one possible origin since the manganese oxide is well known electrocatalyst for water oxidation reaction. Fig. S1 is obtained spinel geometries for the intercalation of H_2O and H_3O^+ at octahedral and tetrahedral sites. For the H_3O^+, we additionally explored the positively charged system (Figure S1e and S1f), in which the Mn_2O_4 framework do not take additional electrons for compensating the intercalated H_3O^+ cation; this can be a chemical intercalation, or charging bias condition, or poor electron conduction case, or the very initial stage of intercalation process. The H_2O and chemically intercalated H_3O^+ prefer the tetrahedral site by -0.13 and -0.28 eV, respectively, while the H_3O^+ with electrons is more stable at the octahedral site by -0.26 eV. This suggests that the H_2O can intercalate into the tetrahedral site, while the H_3O^+ can be located in both tetrahedral and octahedral sites depending on the above mentioned electronic conditions. We note that for all intercalation cases, the interplanar distance of (101) plane is around 5 Å as numbered in Figure S1. Also, we observed that the electrochemically inserted H_3O^+ loses its one H^w at the octahedral site (d_{H2O-H^w} = 1.49 Å); the latter H^w atom then moves close to the Mn_2O_4 framework, forming the OH terminal (Figure S1d).

Details on the Fig.3c. To obtain Fig. 3c, we used total 35 frameworks as follows:

(1 conf.) one initial Mn_2O_4 obtained from the Mn_3O_4 structure, which is referenced as zero.

(4 conf.) four spinel frameworks obtained from $Mn_2O_4(Li)_{0.25n}$ where n = 1, 2, 3, and 4.

(4 conf.) four layered frameworks for Li. (n= 1, 2, 3, and 4)

(8 conf.) four spinel and four layered frameworks for Na (n= 1, 2, 3, and 4 for each)

(8 conf.) four spinel and four layered frameworks for H_2O. (n= 1, 2, 3, and 4 for each)

(8 conf.) four spinel and four layered frameworks for H_3O^+. (n= 1, 2, 3, and 4 for each)

(2 conf.) empty (n=0) spinel and layered frameworks, respectively.

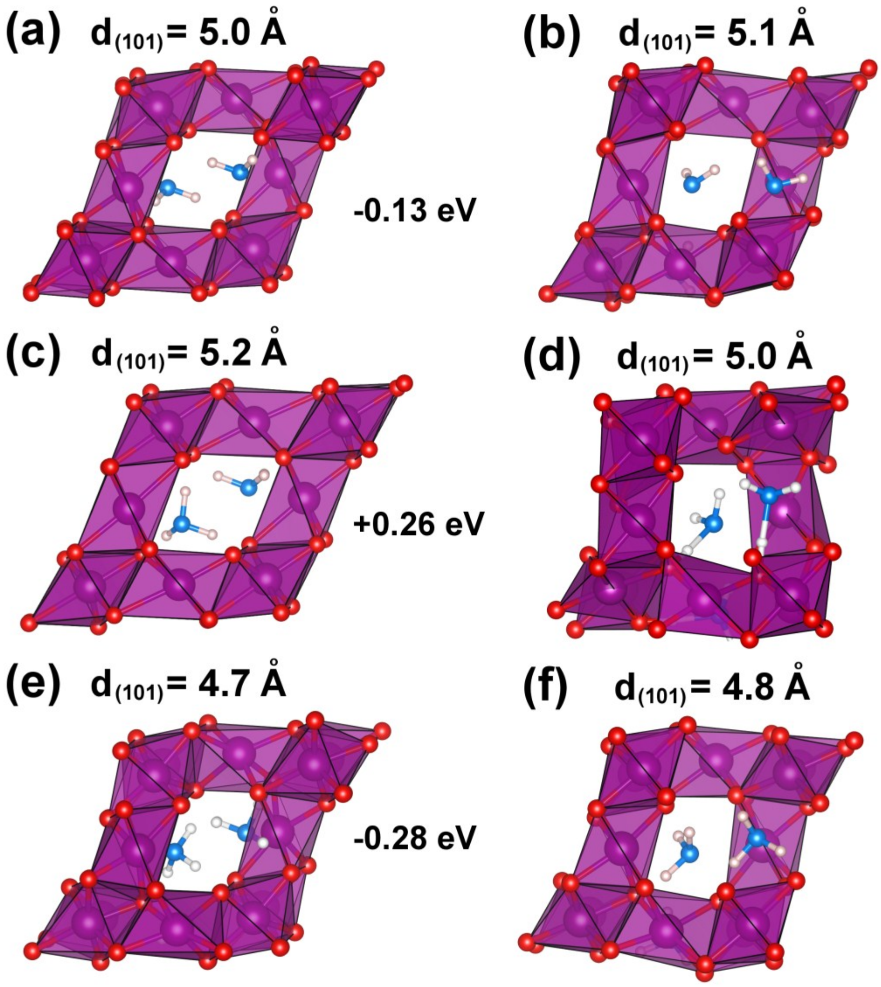

Fig. S1. The intercalation structure of $Mn_2O_4{\cdot}(X)_1$: (a) X = H_2O at tetrahedral site, (b) X = H_2O at octahedral site, (c) electrochemical insertion of X = H_3O^+ at tetrahedral site, (d) electrochemical insertion of X = H_3O^+ at octahedral site, (e) chemical insertion of X = H_3O^+ at tetrahedral site, (f) chemical insertion of X = H_3O^+ at octahedral site. The d values are interplanar distance of (101) planes. Energy values are the relative energy of tetrahedral occupation case compared to each octahedral occupation case (negative value is more stable).

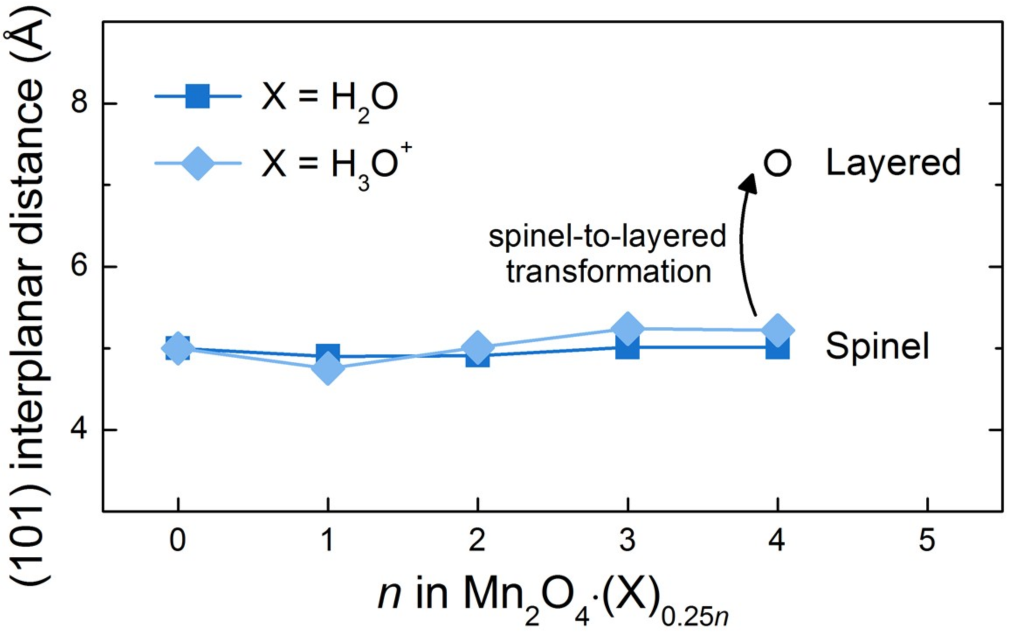

Fig. S2. The (101) plane distance of spinel and layered $Mn_2O_4{\cdot}(H_2O)_{0.25n}$.

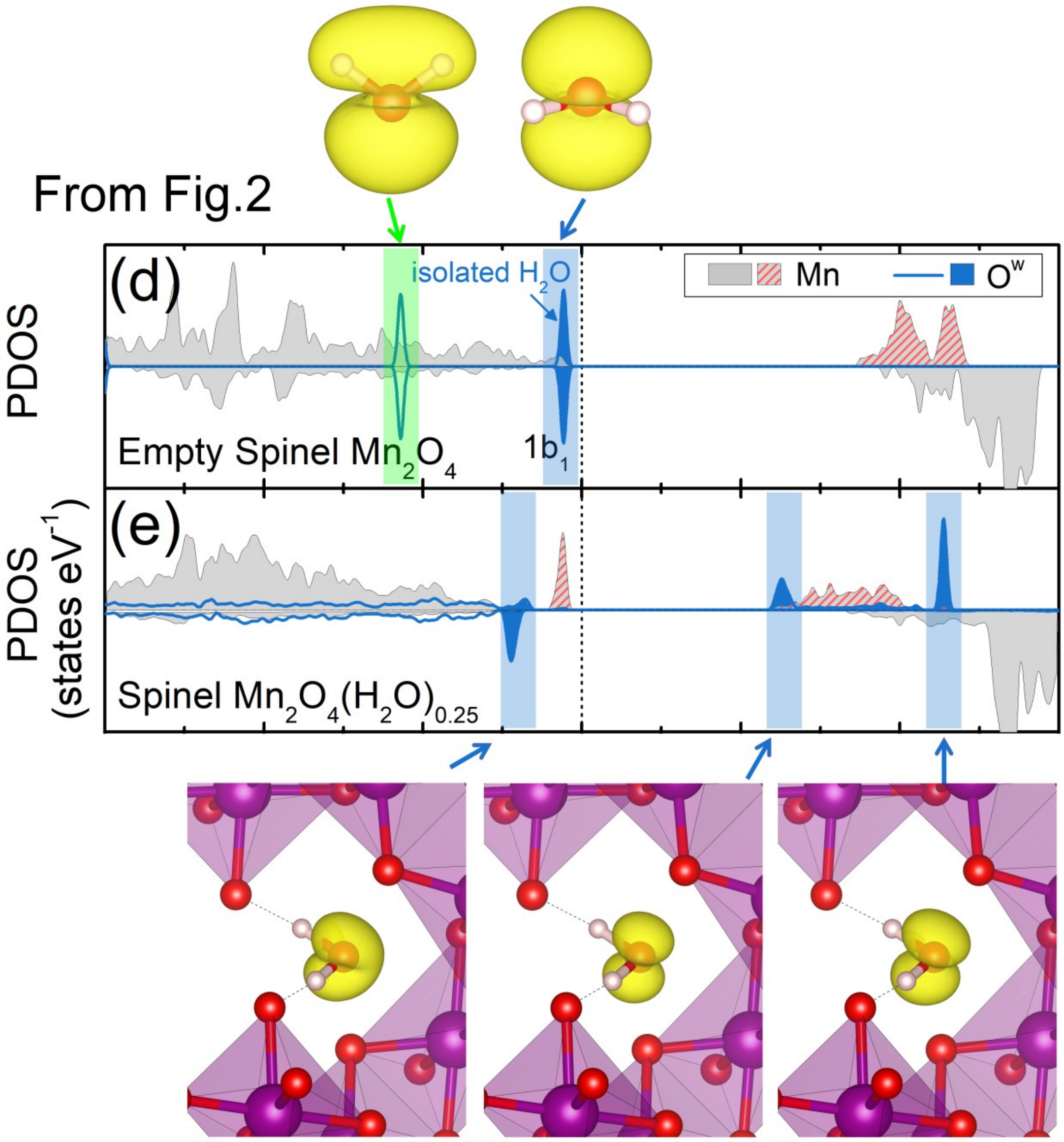

Fig. S3. The electron density isosurfaces which can reflect the shape of orbital. The $1b_1$ orbital is the HOMO of isolated water molecule.

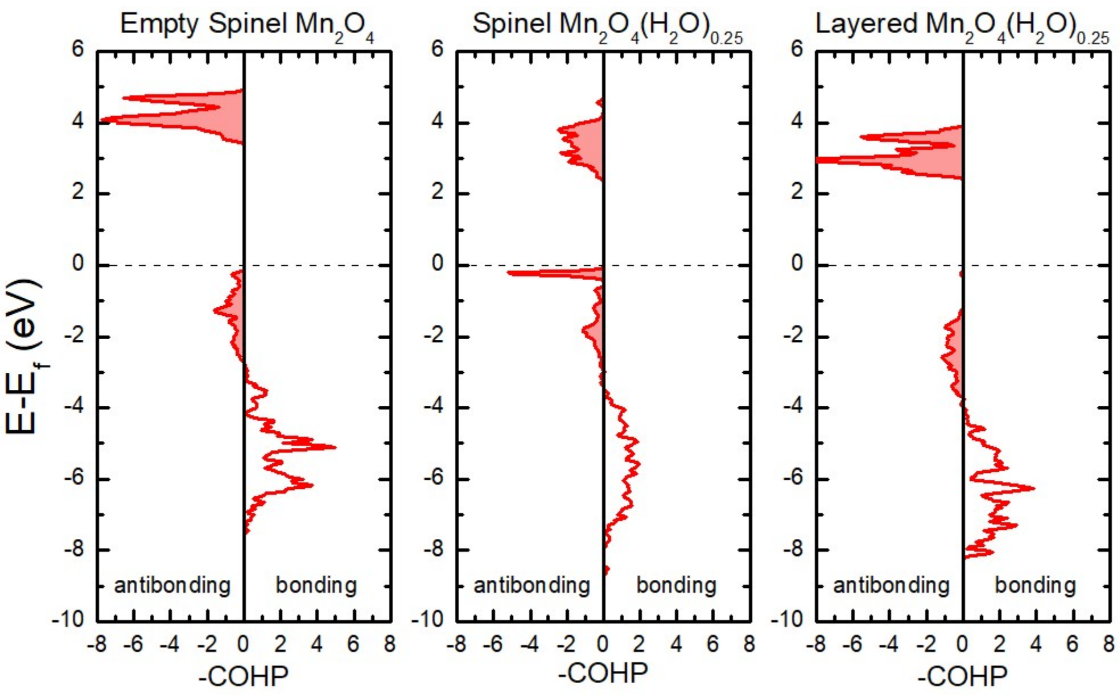

Fig. S4. The crystal orbital Hamilton population (COHP) of up spin electron between Mn center and 6 nearest neighbor oxygen atoms. In the empty spinel Mn_2O_4, states above the Fermi level are antibonding character (filled area). This antibonding orbital is occupied when water molecule intercalates into the spinel phase. PBE0 functional was used.

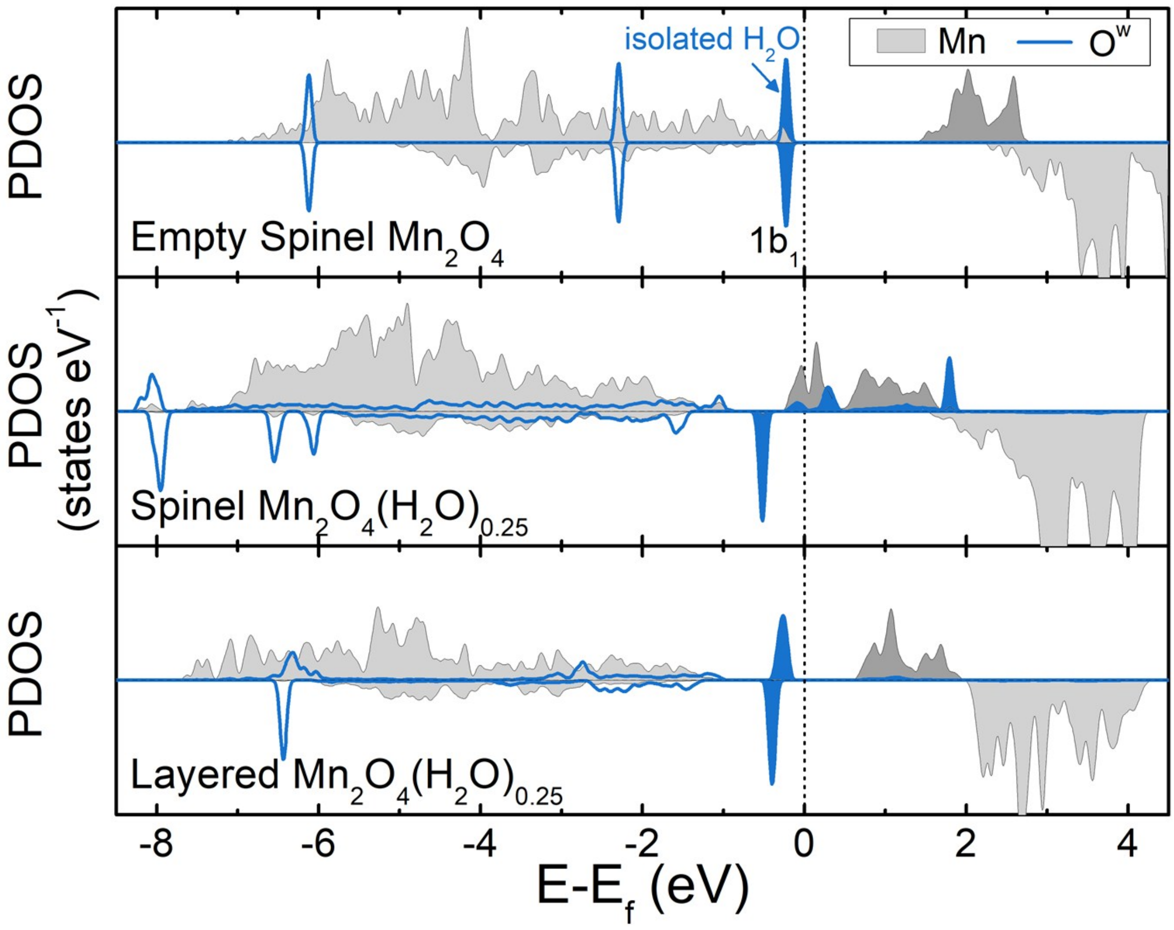

Fig. S5. The PDOS results obtained from GGA+U functional. The band gap is narrower than PBE0 functional, nevertheless, charge transfer phenomena are fundamentally identical to those in PBE0 functional.

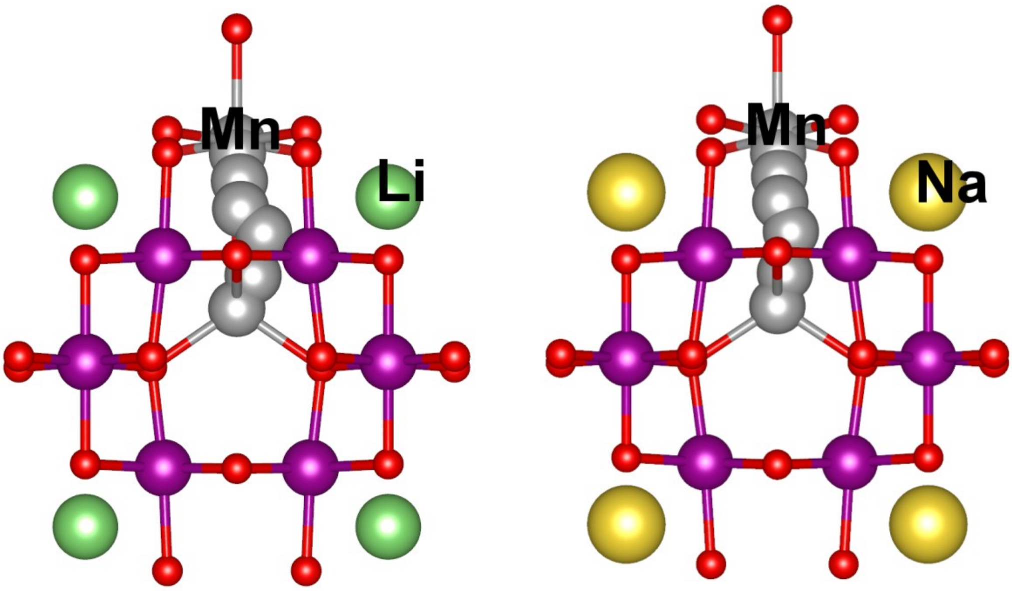

Fig. S6. The Mn migration pathway for Li and Na intercalated spinel structure. The closest distance from traveling Mn to Li (Na) is 2.68 Å (2.93 Å) due to the repulsion between cations. Color codes are identical to the Fig. 4b in the main text.

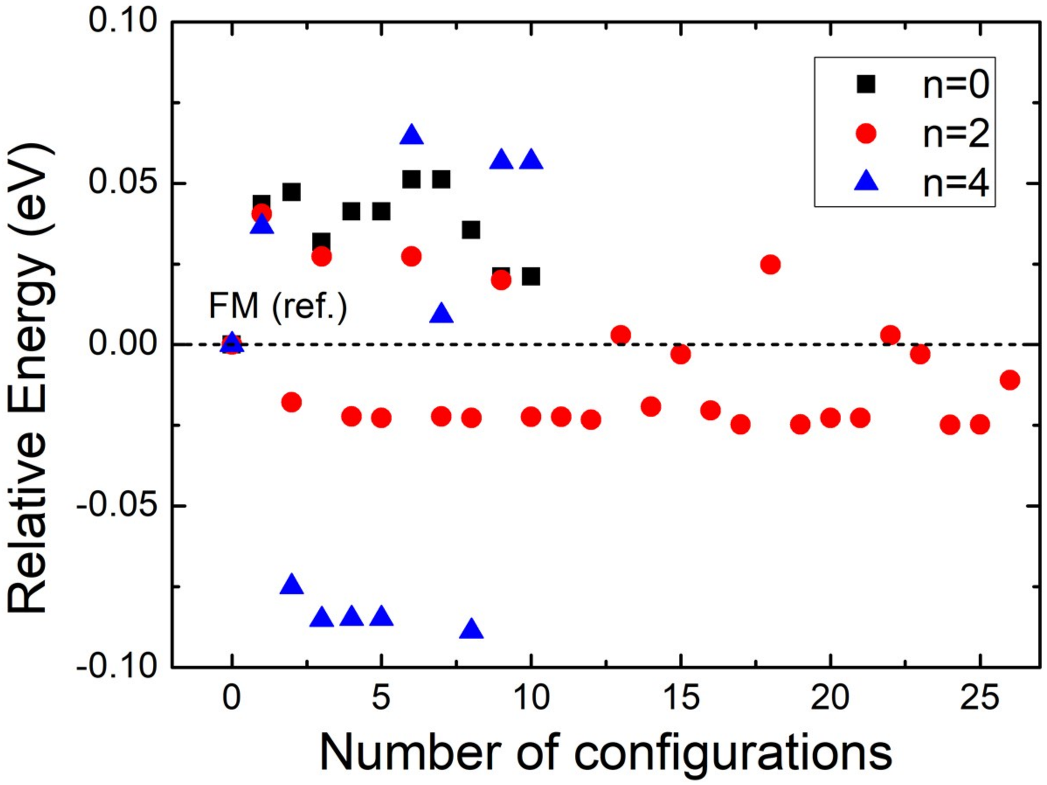

Fig. S7. Relative energy of AFM orderings in reference to the FM ordering. $Mn_2O_4(Li)_{0.25n}$ is used for comparison where n = 0, 2, and 4.

Table S1. Comparison between PBE0 and PBE+U functionals with a dispersion correction of Grimme's D3 method. We performed full relaxation for limited cases due to the computational cost. The $\Delta E_{L\text{-}S}$ tendency (Li > empty > Na) is consistent for both functionals. Lattice parameters of PBE+U were larger than PBE0 by 1–2% constantly.

Structure	Functional	$\Delta E_{L\text{-}S}$ [eV/Mn_2O_4]	Lattice Parameters Difference (PBE+U - PBE0)		
			a [%]	b [%]	c [%]
empty Mn_2O_4	PBE+U	-0.094	2.22 (spinel)<br>2.29 (layered)	2.22 (spinel)<br>1.35 (layered)	2.38 (spinel)<br>1.83 (layered)
	PBE0	-0.102			
$Mn_2O_4(Li)_{1.0}$	PBE+U	0.283	1.87 (spinel)<br>2.06 (layered)	1.87 (spinel)<br>1.38 (layered)	1.83 (spinel)<br>1.09 (layered)
	PBE0	0.363			
$Mn_2O_4(Na)_{1.0}$	PBE+U	-1.110	1.93 (spinel)<br>1.80 (layered)	1.93 (spinel)<br>1.14 (layered)	1.83 (spinel)<br>1.52 (layered)
	PBE0	-1.138			

References.

1. P. E. Blochl, *Physical Review B*, 1994, **50**, 17953-17979.
2. G. Kresse and J. Furthmuller, *Computational Materials Science*, 1996, **6**, 15-50.
3. J. P. Perdew, K. Burke and M. Ernzerhof, *Physical Review Letters*, 1996, **77**, 3865-3868.
4. H. J. Monkhorst and J. D. Pack, *Physical Review B*, 1976, **13**, 5188-5192.
5. S. L. Dudarev, G. A. Botton, S. Y. Savrasov, C. J. Humphreys and A. P. Sutton, *Physical Review B*, 1998, **57**, 1505-1509.
6. L. Wang, T. Maxisch and G. Ceder, *Physical Review B*, 2006, **73**.
7. F. Zhou, M. Cococcioni, C. Marianetti, D. Morgan and G. Ceder, *Physical Review B*, 2004, **70**, 235121.
8. S. Grimme, J. Antony, S. Ehrlich and H. Krieg, *The Journal of chemical physics*, 2010, **132**, 154104.
9. W. Tang, E. Sanville and G. Henkelman, *Journal of Physics: Condensed Matter*, 2009, **21**, 084204.
10. G. Henkelman, B. P. Uberuaga and H. Jónsson, *The Journal of chemical physics*, 2000, **113**, 9901-9904.
